# Unilateral Absence of the Pulmonary Artery Incidentally Found in Adulthood

**DOI:** 10.1155/2012/942074

**Published:** 2012-05-27

**Authors:** Cenk Aypak, Hülya Yıkılkan, Zekai Uysal, Süleyman Görpelioğlu

**Affiliations:** Department of Family Medicine, Dışkapı Yıldırım Beyazıt Teaching and Research Hospital, 06030 Ankara, Turkey

## Abstract

Unilateral absence of the pulmonary artery (UAPA) or pulmonary artery agenesis is a rare congenital disorder presenting with a wide spectrum of symptoms. UAPA is usually associated with cardiac anomalies and surgically treated in childhood. We report a rare case of a 50-year-old woman who was diagnosed with left pulmonary artery agenesis without any other cardiac anomalies. Clinicians should be aware of the possibility of undiagnosed cases of UAPA in patients through a chest radiograph that suggests the diagnosis. Confirmation of the diagnosis and anatomic details can be discerned by CT scanning.

## 1. Introduction

The congenital unilateral absence of the pulmonary artery (UAPA) is a rare anomaly that is frequently associated with other cardiovascular abnormalities. UAPA is estimated to be around 1 in 200,000 individuals [1]. It is thought to be the result of failure in the connection of the sixth aortic arch with the pulmonary trunk during embryologic development [2]. Recurrent pulmonary infections, decreased exercise tolerance, and shortness of breath on exertion are the most common symptoms [1]. UAPA is usually diagnosed and surgically treated in childhood [2]. Most patients who have no associated cardiac anomalies have only minor or absent symptoms and survive into adulthood. These patients give a history of previous consultation with different specialists that results in a variety of erroneous diagnoses, including tuberculosis, Swyer-James syndrome, and lung tumor [2].

In this paper, we describe a case of UAPA without any cardiac anomalies, which was discovered incidentally during periodical examination.

## 2. Case Report

A 50-year-old, nonsmoker woman was admitted to outpatient clinic for periodic examination. She was diagnosed with lung tuberculosis at the age of six and had history of several times of hospitalisation with the diagnosis of recurrent pulmonary infections. She had two term healthy deliveries. She had no complaints.

At the physical examination, slight ipsilateral deviation of the trachea was found. There were fine crackles and decreased breath sounds on the left side. Findings on the rest of the physical examination were unremarkable. Routine hematologic and biochemical profiles were within the normal ranges.

**Figure 1:**
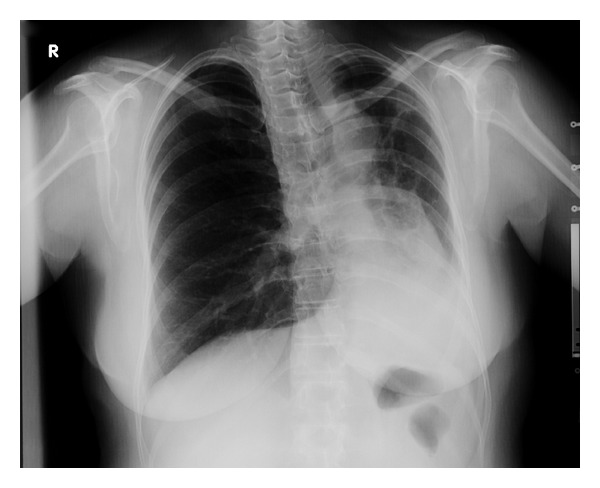
Chest radiograph showing loss of volume of her left lung with displacement of the mediastinum to the left.

Plain radiograph showed a loss of volume of her left lung, cardiac and mediastinal displacement to the left, increased density in the left lower lung zone, and hyperinflation of the lung on the right side (Figure 1). Hemidiaphragm elevation with volume loss of the left lung and absence of hilar shadow were remarkable.

Echocardiogram showed no apparent structural abnormality of the heart, and no pulmonary hypertension (pressure gradient = 20 mmHg) was detected. Pulmonary function tests showed a ratio of forced expiratory volume in one second (FEV1) to forced vital capacity (FVC) of 80%; FEV1 of 2.47 L (61% of predicted), FVC of 2.90 L (52% of predicted), total lung capacity of 67% of predicted, and a diffusion capacity for carbon monoxide of 72% of predicted.

**Figure 2:**
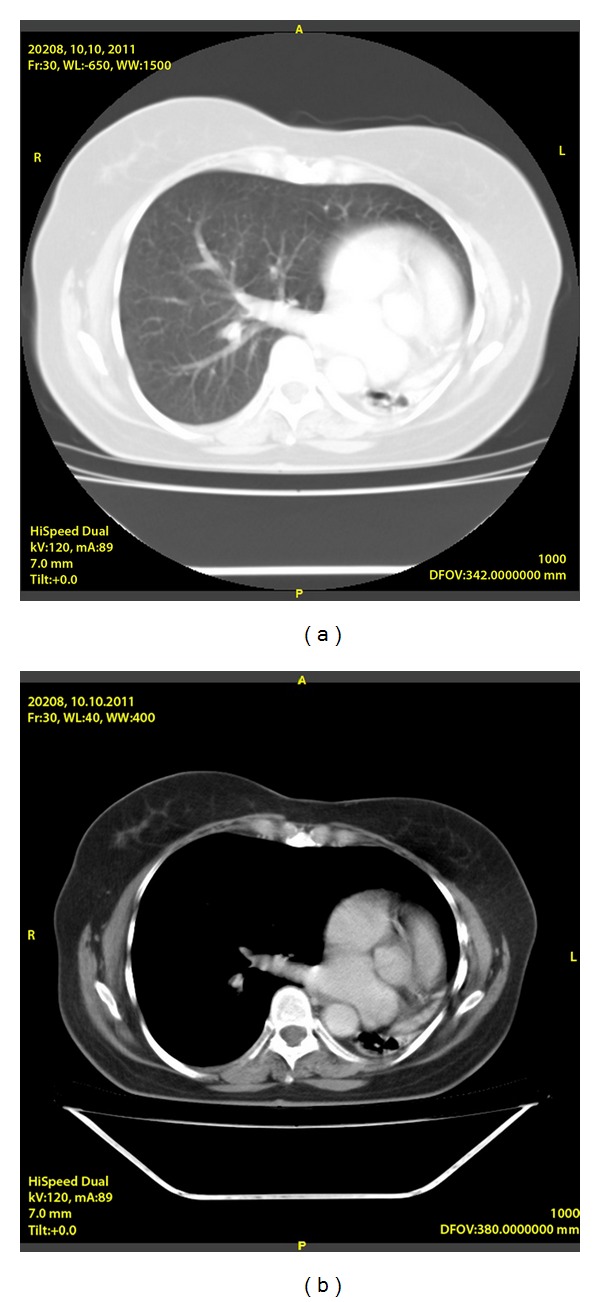
Thoracic CT scan revealing absence of left pulmonary artery and ipsilateral pulmonary hypoplasia.

Contrast-enhanced 7 mm collimator computed tomographic (CT) of the chest showed an enlargement of the pulmonary artery trunk and demonstrated the absence of the left pulmonary artery with displacement of heart and mediastinum to the left and volume loss associated with increased interstitial markings involving her left lung (Figures 2(a) and 2(b)). Cardiac arteriographic study revealed no other additional cardiac abnormalities.

We decided to follow up the patient for respiratory symptoms, such as breathlessness, hemoptysis, and pulmonary hypertension. The risk factors regarding the absence of a pulmonary artery and travelling to high altitude were explained to our patient. Her clinical status remained stable, without any symptoms, during the follow-up period.

## 3. Discussion

UAPA was first described in 1868 [1]. Left-sided agenesis seems to be more frequently associated with cardiac abnormalities, and therefore early diagnosis and surgical repair are required during the first year of life [1, 2]. Conversely the patient in this paper had left-sided UAPA and had no other cardiovascular anomalies. Patients with isolated right UAPA survive into adulthood with minimal symptoms, making the diagnosis of such cases more difficult. Symptoms can sometimes be provoked by factors such as pregnancy or high altitude [2]. Although our patient had two term deliveries, she had no complaints.

The diagnosis of UAPA is difficult, especially when chest radiographic abnormalities are first noted in adulthood. The diagnosis of UAPA is generally based on medical history, physical examination findings, and the results of chest radiographs [3]. Typical radiographic findings are ipsilateral cardiac and mediastinal displacement, ipsilateral hemidiaphragm elevation with volume loss of the affected lung, absent hilar shadow, and hyperinflation of the contralateral lung. Our patient had history of several times of hospitalisation with the diagnosis of recurrent pulmonary infections. The abnormal findings in her chest radiograph were interpreted as a result of the pulmonary infections.

Echocardiography is a good tool to establish the diagnosis and to evaluate the presence of associated pulmonary hypertension. CT and MRI were found to be useful modalities for accurate anatomic depiction of UAPA [3, 4]. Both techniques enabled UAPA to be distinguished from acquired obstruction of the pulmonary artery. Angiography remains the gold standard for the diagnosis of pulmonary artery agenesis but currently it is rarely performed with the development of CT and MRI and just considered for the patients who need surgical intervention [5, 6].

Regarding the treatment of UAPA, it is reported that 8% of the patients underwent either a pneumonectomy or a lobectomy for recurrent hemoptysis or intractable pulmonary infections and 7% of the patients underwent revascularization of hidden pulmonary arteries [2]. It is necessary for these patients to be followed up closely, especially for the observation of their pulmonary hemodynamics.
